# Affinity probes based on small-molecule inhibitors for tumor imaging

**DOI:** 10.3389/fonc.2022.1028493

**Published:** 2022-10-27

**Authors:** Xinzeyu Yi, Zheng Wang, Xiang Hu, Aixi Yu

**Affiliations:** Department of Orthopedics Trauma and Microsurgery, Zhongnan Hospital of Wuhan University, Wuhan, China

**Keywords:** inhibitor, affinity probe, near-infrared, radiotracer, tumor imaging

## Abstract

Methods for molecular imaging of target areas, including optical imaging, radionuclide imaging, magnetic resonance imaging and other imaging technologies, are helpful for the early diagnosis and precise treatment of cancers. In addition to cancer management, small-molecule inhibitors are also used for developing cancer target probes since they act as the tight-binding ligands of overexpressed proteins in cancer cells. This review aims to summarize the structural designs of affinity probes based on small-molecule inhibitors from the aspects of the inhibitor, linker, dye and radionuclide, and discusses the influence of the modification of these structures on affinity and pharmacokinetics. We also present examples of inhibitor affinity probes in clinical applications, and these summaries will provide insights for future research and clinical translations.

## Introduction

Multiple enzymes and receptor proteins in organisms are involved in life processes such as cell metabolism, proliferation, differentiation, migration, and apoptosis by regulating biochemical reactions or signaling pathways. Small-molecule inhibitors can regulate protein function by reversibly or irreversibly binding with these proteins (1, 2). By specifically binding to highly expressed proteins in cancer cells and producing effects, many small-molecule inhibitors have been used in targeted cancer therapy. Moreover, new targets and subtype-selective inhibitors have also been developed in response to the problems of cancer resistance and potential side effects (3–6). On this basis, affinity probes based on small-molecule inhibitors (AfPIs) for targeted cancer imaging have become research areas of major interest in recent years. Despite their severe metabolic problems, like peptide probes (7, 8), AfPIs not only have the advantages of non-immunogenicity, easy structure modification, fast target recognition, and strong affinity, but also have a broader biodistribution and a higher signal-to-noise ratio than antibody-conjugated probes or peptide probes (9, 10). Hence, they are efficient tools for cancer research and have broad application prospects in early diagnosis, prognosis assessment, surgery navigation and drug delivery monitoring (11, 12).

**Scheme 1:**
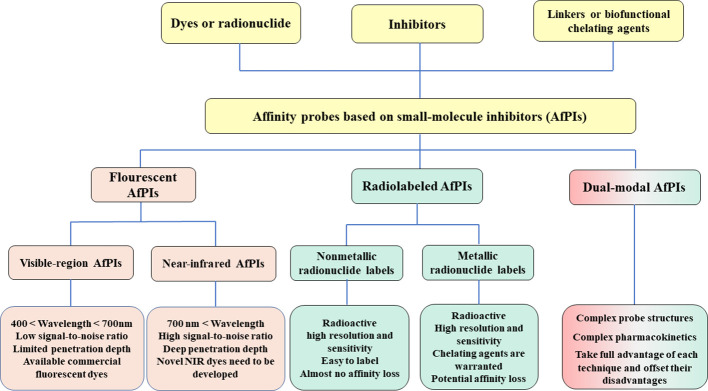
The classifications of AfPIs and their features. In this review, we classified AfPIs into visible-region, near-infrared, radiolabeled and dual-modal probes, and introduced them from three aspects: the *inhibitors, linkers* and *dyes* or *radionuclides*.

This review summarizes the tumor-targeting AfPIs emerging in recent years and aims to provide design strategies for developing novel AfPIs. The key challenges and corresponding solutions in the design of such probes are discussed below. Herein, we classify AfPIs into traditional visible-region, near-infrared, radiolabeled and dual-modal probes for comparison. We specifically focus on near-infrared and radiolabeled probes with promising clinical applications, and reveal the characteristics of the two probe types and provide references for future clinical translation. **Scheme 1** summarises the classifications of AfPIs and their features.

## Visible-region AfPIs

**Figure 1:**
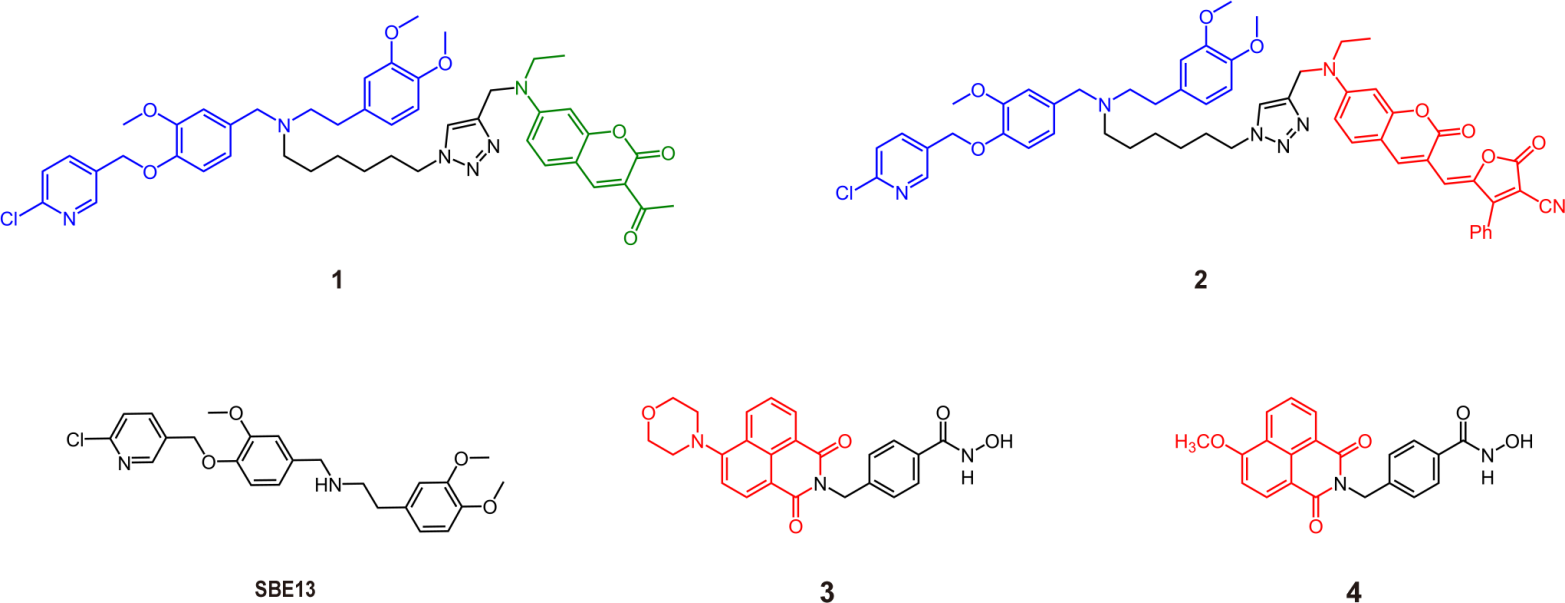
Some structures of traditional visible AfPIs and their parent inhibitors (Blue, inhibitor structure; green and red, fluorophores).

Fluorescence imaging is an excellent, noninvasive imaging method that allows the visualization of cell status and many biochemical reactions (13, 14). The introduction of inhibitor structures enhances the targeting ability of probes to distinguish cancer from the normal region. This section focuses on the fluorescent AfPIs in the visible region (wavelength below 700 nm), mainly used for targeted imaging of cells or tissues. As shown in **Figure 1**, the recognition group Polo-like kinase 1 (PLK1) inhibitor **SBE13** (15) was conjugated with linker and coumarin derivatives chosen for fluorophores, forming two kinds of PLK1 affinity probes, **1** and **2**, with emission wavelengths of 480 nm and 660 nm, respectively. Modifying the coumarin structure in **2** resulted in intramolecular charge transfer (ICT), and a redshift close to the near-infrared region in its emission could be imaged *in vivo* (9). Overexpression of PLK1 in some human tumor cells makes it a target for antitumor drug treatment (16). By binding with PLK1, the probe is concentrated in the PLK1 kinase-rich region to distinguish it from normal regions. Although the imaging effect of **2** was demonstrated *in vivo*, 660 nm is insufficient to meet the needs of *in vivo* detection. In addition, inhibitors with unique structures can also serve as fluorescent moieties; hence, no extra dye conjugation is warranted. For instance, histone deacetylase 6 (HDAC6) inhibitors containing a naphthalimide skeleton, which is intrinsically fluorescent, were synthesized as inhibitor-based affinity probes (**3** and **4**) to detect the expression of HDAC6 in tumor cells (**Figure 1**) (17, 18). Moreover, there are affinity probes based on the biotin-avidin system that conjugate inhibitors and biotin for proteomic analysis and imaging in cells (19). However, these probes without an OFF-ON function will lead to false positives and phototoxicity because they will be retained in normal tissue regions and release fluorescence. Furthermore, their low signal-to-noise ratio (SNR) blurs the tumor location (20, 21).

**Figure 2:**
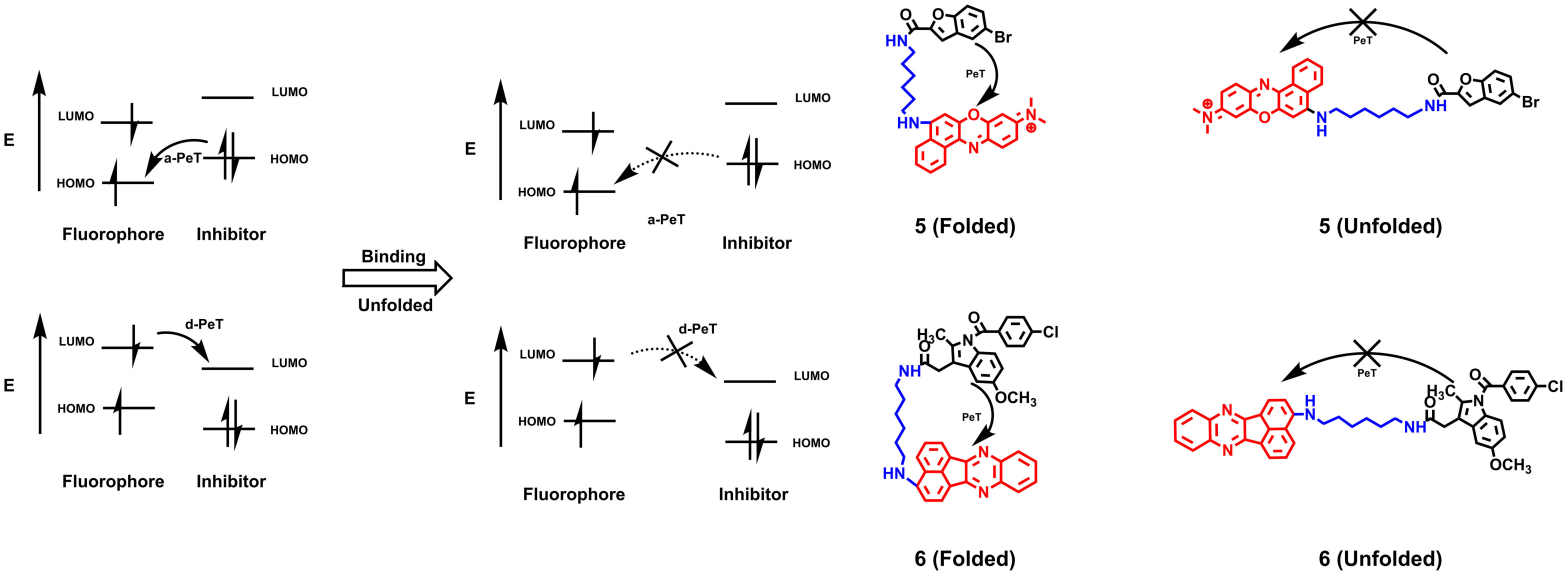
The quenching mechanism of PeT effects and AfPIs are designed based on PeT effects. When probes do not bind to the proteins, the fluorescence is quenched by Pet effects. After binding to proteins, the folded structure is open and the PeT effect disappears, resulting in the release of fluorescence (HOMO, highest occupied molecular orbital; LUMO, lowest unoccupied molecular orbital).

Hence, smart probes with an “OFF-ON” design appear more attractive. Because affinity probes bind to proteins directly, the “trigger” can be activated by changing the spatial conformation rather than an enzymatic or chemical reduction stimulus (22). Photoinduced electron transfer (PeT) involves a-PeT and d-PeT processes. In the a-PeT process, the inhibitor provides electrons to the highest occupied molecular orbital (HOMO) of the adjacent fluorophore. In contrast, the fluorophore donates its electrons to the lowest unoccupied molecular orbital (LUMO) of the inhibitor in the d-PeT process. Finally, the electrons in the LUMO of the fluorophore fail to return to the HOMO, resulting in fluorescence quenching (23). When the inhibitor binds to the target, changes in the spatial structure or electronic energy levels will disrupt the process, releasing fluorescence (**Figure 2**). Based on this principle, Peng et al. (24) used the intramolecular spatial folding effect caused by small-molecule inhibitors and dyes to design the fluorescence probe **5** targeting the Golgi apparatus of cancer cells based on the cyclooxygenase 2 (COX-2) inhibitor indomethacin (IMC). When IMC binds to the amino acid residues Arg120, Tyr355 and Glu522 of the COX-2 molecule, its folded structure is open, and the PeT effect disappears, resulting in the release of fluorescence with a maximum excitation wavelength of 547 nm. Although the two-photon property of the probe has improved its tissue penetration to a certain extent, its emission wavelength still limits its application in biological imaging *in vivo*. Based on 5-bromobenzofuran-2-carboxylic acid, an inhibitor of Pim-1 kinase, Guo designed probe **6** with a PeT effect, whose emission wavelength reached the red light level and achieved live animal imaging of tumor xenograft mice (25). Similar to the COX-2 probe, probe **6** changes from the folded state to the unfolded state by binding with Pim-1 kinase, thereby removing the fluorescence quenching and releasing the fluorescence. Compared with traditional non-OFF-ON probes, this type of probe utilizes the conformational changes of inhibitors and dyes to exhibit a higher SNR, reduce the phototoxicity of nontargeted areas, and significantly reduce the false-positive phenomenon during imaging.

Many commercial fluorescent dyes in the visible region have been developed (26), and less steric hindrance and better pharmacokinetics can be easily obtained by modifying the structure of dyes. However, due to the short wavelength of these probes, it is difficult to obtain good results for *in vivo* imaging, so they are more suitable for qualitative or semiquantitative research at the molecular level and imaging at the level of cells or tissue slices. Designing near-infrared probes with near-infrared dyes is the future trend in the clinical translation of AfPIs.

## Near-infrared AfPIs

The near-infrared (NIR) band can be roughly divided into near-infrared window I (700-1000 nm) and near-infrared window II (1000-1700 nm) (27) and exhibits a higher penetrating capability than visible fluorescence in tissues. In addition, compared with traditional visible-light imaging, near-infrared imaging is less affected by biological matrix scattering and tissue autofluorescence, which gives it a higher signal-to-noise ratio and better spatial resolution. Therefore, near-infrared imaging is more suitable for *in vivo* imaging, and NIR AfPIs are also ideal for early diagnosis, surgery navigation and photothermal therapy of tumors (28–30). Near-infrared inhibitor probes mainly include three structures: inhibitors, linkers and near-infrared dyes. The influences of these three structures on the affinity and metabolism of the probe and the design strategy of the probe are discussed in the following.

### Inhibitor structure in AfPIs

**Figure 3:**
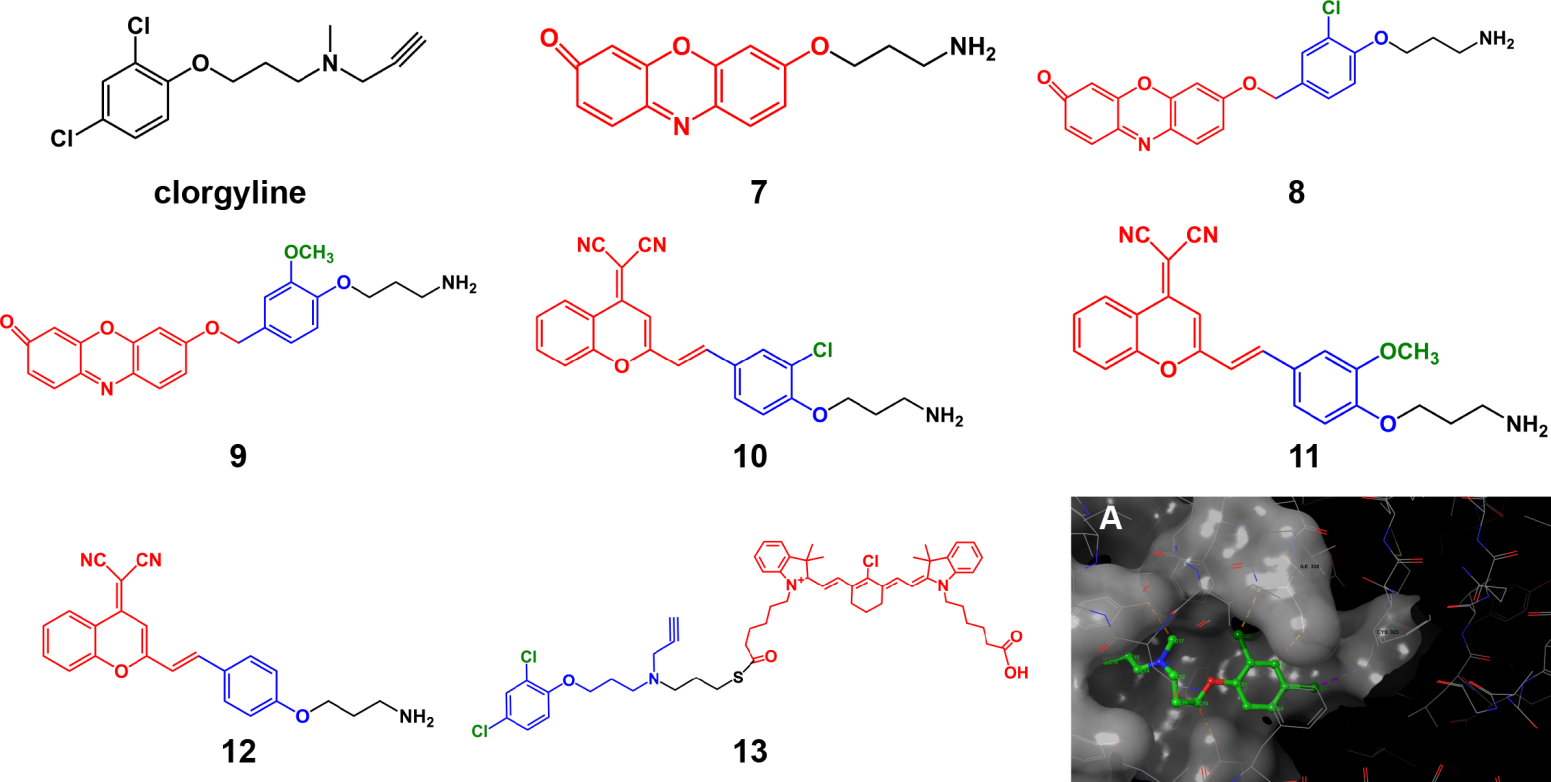
Structures of clorgyline and clorgyline-derived AfPIs (Blue, inhibitor structure; red fluorophores). **(A)** Molecular docking shows that clorgyline forms hydrogen bonds between its chlorine atoms and the Cys323 and Thr326 residues of MAO-A (PDB ID: 2BXR).

The presence or absence of the inhibitor structure in the probe and the modification of crucial groups in the inhibitor structure will significantly impact the probe’s affinity and selectivity. Taking the monoamine oxidase (MAO) series of probes as an example, MAO is an important enzyme that regulates some biochemical reactions in the body, controlling the metabolism of catecholamines and serotonin. It plays a crucial role in the progression of tumors and Parkinson’s disease. MAO contains two isoforms: MAO-A and MAO-B. The original design of the MAO-A targeting probe **7** only contains a fluorophore and propylamine group as the recognition moiety. When propylamine meets MAO, the propylamine group undergoes a continuous oxidation/β-elimination reaction and is removed, releasing free fluorescent groups and producing fluorescence (31). However, this probe shows no subtype selectivity and has insufficient affinity. Based on this probe structure, Wu et al. (32) introduced the structure of the MAO-A selective inhibitor clorgyline to the probe (**8**), which gave the probe higher MAO-A affinity and selectivity. Replacement of the chlorine substituent on the benzene ring, such as the methoxy group (probe **9**), drastically decreased the selectivity of the probe to lower than that of **8** but still higher than that of the previous generation probe **7**, which lacked an inhibitor structure. Similarly, the clorgyline derivative probes **10** and **11** based on the dicyanomethylene-4H-pyran chromophore (DCM) structure developed by Yang et al. (33) had a higher selectivity for MAO-A than MAO-B, with relative fluorescence intensity of approximately 42-fold. However, when the halogen substituent was changed, the affinity of the unsubstituted (H atom) probe **12** decreased slightly, and the selectivity decreased by approximately 20-fold. The resulting product lost selectivity and affinity if it was substituted with methoxy or methyl. Comparing the performance of these probes shows that in addition to the fact that the halogen element chlorine plays a key role in binding, steric hindrance may also have a certain effect. This potential effect is consistent with previous molecular docking results for MAO-A and clorgyline (34). When clorgyline undergoes docking with MAO-A, two chlorine atoms form hydrogen bonds with the Cys323 and Thr326 residues of MAO-A (**Figure 3A**). These hydrogen bonds help stabilize the binding between the inhibitor and the protein. Wu et al. (35) chose to connect the NIR dye to the other end of the clorgyline to synthesize **13**, protecting two chlorine atoms so that the probe had a more potent antitumor ability than the parent compound. Although the mitochondrial-targeting effect of the NIR dyes here contributes to the antitumor ability, it also illustrates the importance of protecting key groups.

The above studies indicate that the interaction between the targets of some key groups of AfPIs and the steric hindrance of some groups play critical roles in the performance of probe affinity. In designing AfPIs, the groups of the inhibitor that play a vital role in binding to the target must be protected to avoid diminishing the overall affinity of the probe. However, the loss of certain key groups does not necessarily or directly lead to the failure of probe imaging. For example, in the aforementioned OFF-ON probe based on the Pim-1 inhibitor, the carboxyl group on its parent inhibitor structure can form a salt bridge and hydrogen bond with Pim-1 kinase, which is crucial for binding kinase. And when the carboxyl group is destroyed, this will lead to an apparent loss of affinity (36). This result shows that imaging can still be achieved in the case of the loss of some key groups, possibly because the benzene ring still contains a bromine atom to help stabilize the binding, and the OFF-ON imaging mechanism avoids the fluorescence of probes when they are not bound to the kinase. This also illustrates the imaging advantages of OFF-ON probes from another aspect, which can avoid the problem that the tumor cannot be distinguished sufficiently from the surrounding normal tissues due to a loss of affinity.

**Figure 4:**
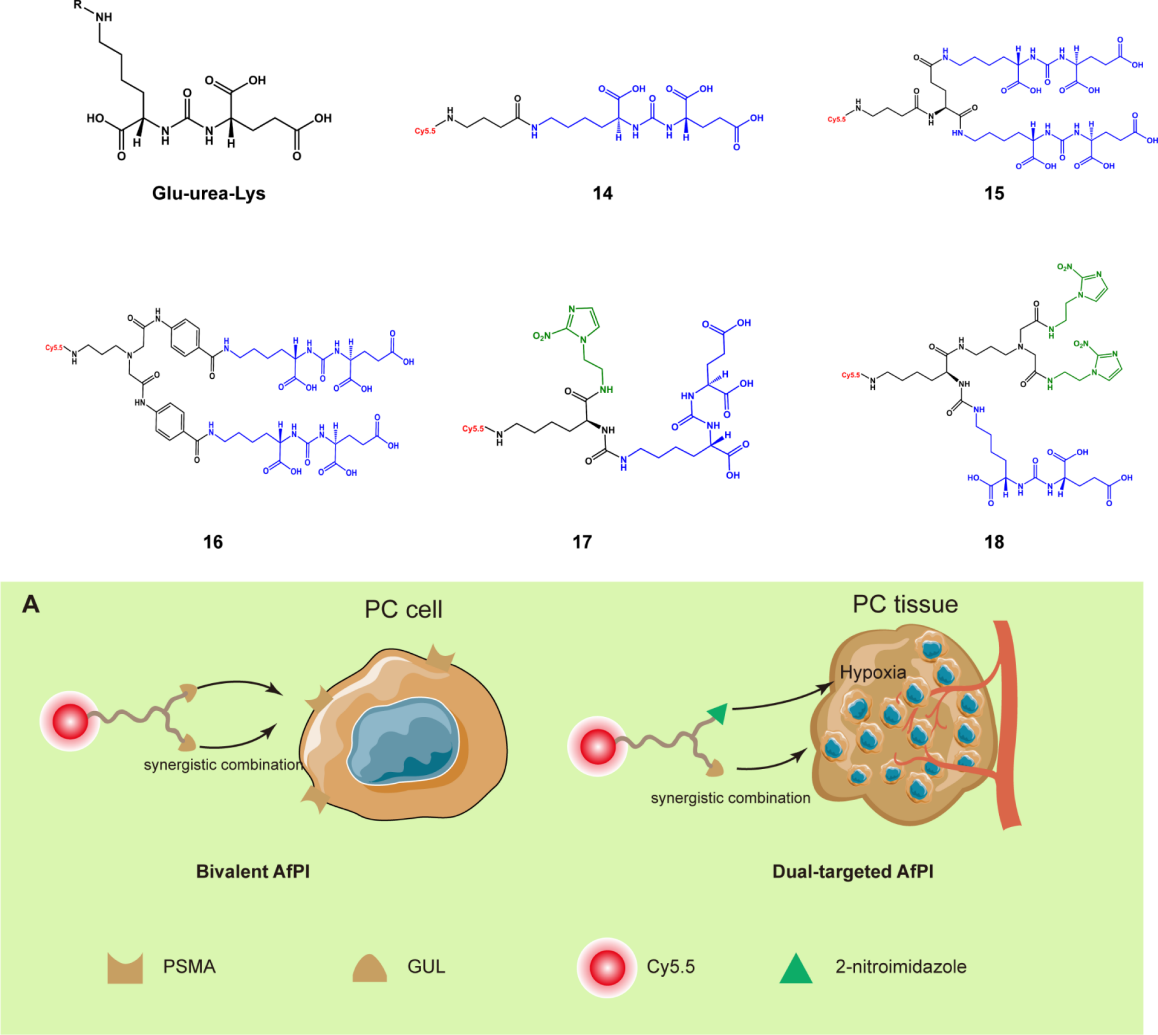
Structures of the PSMA inhibitor and its derived AfPIs (Blue, GLU units Green, 2-nitroimidazole group). **(A)** Schematic of bivalent and dual-targeted AfPIs for prostate cancer (PC, prostate cancer).

Containing multiple inhibitor structures or co-targeting through multiple regions can also help probes more easily gather in the target region. Prostate-specific membrane antigen (PSMA), a peripheral glutamate carboxypeptidase, is a biomarker highly expressed by prostate cancer cells. PSMA is located on the cell membrane surface, and its active site faces the outside of the cell; this enzyme has become a common target for AfPIs (37). Its representative inhibitor structure is glutamate-urea-lysine. Based on this structure, the NIR dye can be connected to achieve targeted prostate cancer imaging (38, 39). On this basis, Kwon et al. (40) established two bivalent AfPIs, **15** and **16,** with two GLU units, and these probes exhibited a higher tumor uptake rate than that with only one GLU unit (**14**). Later, 2-nitroimidazole, which has a targeted hypoxia effect, was introduced onto the other end of the structure to synthesize **17** (41) so that the dual-targeting effect of hypoxia and PSMA was achieved without significant loss of the original affinity of PSMA(**Figure 4A**). There was a partial loss of affinity in compound **18** with the introduction of two 2-nitroimidazole groups simultaneously, which may be ascribed to the increased steric hindrance. The simultaneous existence of multiple recognition groups further enhances the imaging effect, reducing the false-negative rate and thus identifying tumor regions more clearly. At the same time, attention needs to be paid to the increase in steric hindrance caused by introducing new groups.

**Figure 5:**
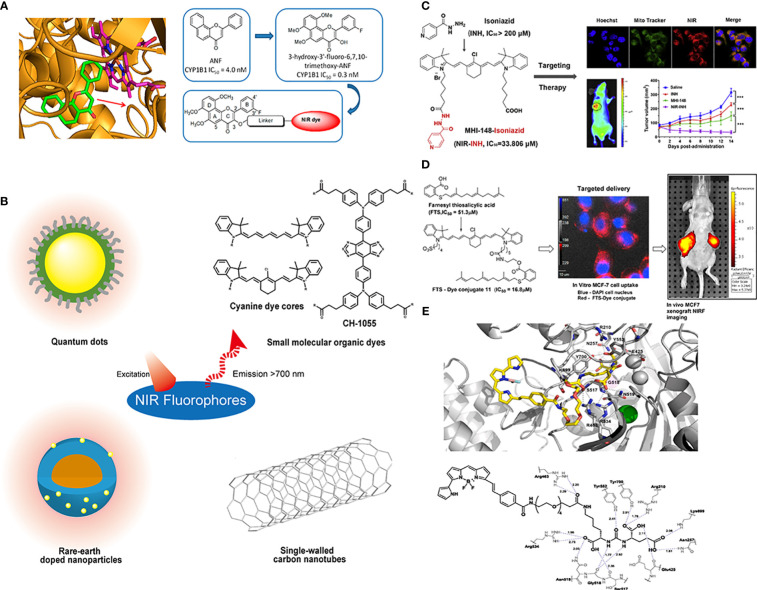
Conjugations between inhibitors and NIR fluorophores. **(A)** Conducting molecular docking analysis of CYP1B1 inhibitor and its target before conjugation to avoid the loss of affinity. Reprinted with permission (42). Copyright 2018 American Chemical Society. **(B)** The common types of NIR fluorophores. **(C)** Conjugating with the heptamethine cyanine dye MHI148 can improve the antitumor effect of the MAO-A inhibitor isoniazid. Reprinted with permission (43). Copyright 2018 Elsevier. **(D)** The conjugation of FTS with cancer-targeting heptamethine cyanine dye improved its pharmacological profile. Reprinted with permission (44). Copyright 2017 American Chemical Society. **(E)** Molecular docking results demonstrated a 20 Å tunnel region in PSMA. Reprinted with permission (45). Copyright 2020 Elsevier.

When designing a novel AfPI, the factors affecting inhibitor affinity must be considered, and the probe should be designed as a new “inhibitor”. For example, when designing the structure of CYP1B1 targeted AfPI, Meng et al. (42) excluded areas bound to the enzyme and made modifications in a relatively safe area *via* molecular docking (**Figure 5A**). Wang et al. (46) avoided the sulfonamide structure of celecoxib and chose to modify the pyrazole ring position to reduce the loss of affinity. In this approach, determining the inhibitor’s crucial structure, attempting to protect these structures in connecting dyes and linkers, and performing molecular dynamics simulation on these structures is conducive to predicting whether the synthesized probe can bind to the target protein.

### Dyes and linkers in AfPIs

Near-infrared dyes can be roughly classified into two types: nonorganic and organic. Nonorganic dyes include single-walled carbon nanotubes, quantum dots, and rare-earth materials (26) (**Figure 5B**). Similar to antibodies, inhibitors can be introduced into these inorganic dyes through covalent or noncovalent binding to achieve targeted imaging (47, 48), in which covalent binding is more stable, and these inorganic materials can also be used to deliver targeted drugs to achieve the integration of diagnosis and treatment (49). However, these inorganic materials need to be functionalized in advance (50), and the limitations of water solubility, photothermal stability, immunity uptake and biological clearance in the body must be addressed (51).

Organic dyes have lower molecular weight and higher plasticity, biocompatibility and safety than inorganic dyes. Additionally, some of them have been approved for clinical use or have started in clinical trials, such as indocyanine green (ICG). Some heptamethine cyanine dyes can also preferentially accumulate in the mitochondria of tumor cells through the high expression of organic anion transporter peptides (OATPs) in tumors and the higher transmembrane potential of tumor cells (52, 53), and they can achieve tumor seeking, accumulation and retention *via* the covalent binding of *meso*-chlorine and albumin (54). The conjugation of these dyes and small-molecule inhibitors provides a way to change the pharmacokinetics (55). In addition, the overall properties of organic dyes, such as excitation/emission wavelengths, water-solubility and photostability, can be easily adjusted by chemical modification.

The introduction of dyes and linkers is related to the affinity and pharmacokinetics of the probe, and the differences in some substituents on these dyes will alter the probe metabolism and accumulation of the tumor area. Generally, when choosing dyes, better water solubility and higher emission wavelengths are pursued because these characteristics will be conducive to clinical translation. However, in the process of conjugating dyes, due to steric hindrance or changes in functional groups, the overall affinity of the probe will decrease, which is not conducive to later targeted imaging. Therefore, suitable dyes and synthetic routes should be chosen to avoid loss of affinity. Additionally, better imaging results can be achieved if improvements can be made to synthesize probes that overcome parent inhibitors’ deficiencies. Genistein has limited clinical antitumor applications because of its low oral bioavailability and poor pharmacokinetics. Guan et al. (56) conjugated genistein with the near-infrared dye IR-783 and used the advantage that IR-783 could be transported by OATPs and enriched in breast cancer cells to improve its antitumor effect and achieve NIR imaging. Similarly, Lv et al. (43)conjugated the MAO-A inhibitor isoniazid with the heptamethine cyanine dye MHI148 and used its mitochondrial targeting ability to obtain a theranostic probe for prostate cancer (**Figure 5C**), which showed a more potent antitumor effect than the parent inhibitor isoniazid. Similar designs have been reported in many other studies. When S-trans-trans-farnesyl salicylic acid (FTS), an RAS and mammalian target of rapamycin (mTOR) inhibitor, was connected with the heptamethine cyanine dye, the inhibitory effect on mTOR and antitumor effect of the probe was better than FTS, and the EC_50_ was reduced from 51.3 nm to 16.8 nm (**Figure 5D**) (44). These results may be ascribed to the fact that the sulfonate group and the tumor-targeting ability of the dye improve its dose distribution. Inorganic dyes can also achieve this effect. Hu (7) combined carbon quantum dots with an ST14 (suppressor of tumorigenicity 14) inhibitor to improve renal clearance and increase the retention of the inhibitor in tumors, which is beneficial for its antitumor effect and imaging. These studies demonstrated that the improved pharmacokinetics ascribed to introducing dyes and linkers could enhance the tumor targeting and antitumor ability of AfPIs.

Although there have been many studies on AfPIs in the first NIR window, AfPIs whose emission wavelength falls in the second NIR window are just emerging (57, 58), and the wavelength of the existing inhibitor probes is generally low, possibly because it is relatively difficult to design novel dyes. To achieve a redshift of the wavelength, extended π conjugation is required (59). After the probe is combined with small molecules, the resulting structure will become more complex, and the binding effect will be more uncontrollable, so existing dyes are conjugated in most studies. Furthermore, when the wavelength of dyes redshifts to the second NIR window, their quantum yields drop sharply (60). Other issues that NIR dyes share, including water solubility and probe biocompatibility, are challenges that still need to be overcome in studying inhibitor NIR-II window probes.

The linker is also critical to the properties of the probes. It can avoid the effect of steric hindrance of the dye on the affinity of the inhibitor and can improve the metabolic kinetics of the probe through modifications, such as with polyethylene glycol (PEG). Taking prostate cancer as an example, Son et al. used the PEG chain as a linker to conjugate 4,4-difluoro-4-bora-3a,4a-diaza-*s*-indacene (BODIPY) and Glu-CO-Lys to construct probes (45). The molecular docking results showed that the PEG linker was located in the tunnel region, with a length of approximately 20 Å (**Figure 5E**), which is consistent with previous findings (61). This design allows the entire fluorophore molecule to be outside the target molecule and avoids steric hindrance caused by the introduction of the bulky dye. PEG improves the water solubility and biocompatibility of the probe and eliminates adverse effects of the lipophilic dye BODIPY so that its metabolic kinetics *in vivo* are improved, and the overall affinity is also ensured. When Kwon et al. (40) attempted to change the glutamine structure in the linker to a benzene structure to obtain **16** based on the structure of **15**, the probe showed slower clearance and lower affinity than **15** because of the introduction of the benzene ring structure on the linker. The same is true for the principle of designing radionuclide probes and modifications in the linker area can significantly improve the tumor uptake rate and *in vivo* pharmacokinetics (37, 62).

### Clinical applications

The probe tool should be based on actual clinical problems and converted into clinical applications, which is our ultimate goal in designing AfPIs. Zhu et al. (63) used two AfPIs to perform dual-target imaging of BCL2 and MDM2, simultaneously detecting the activities/expression of apoptosis markers. Arlauckas et al. (64) designed and synthesized JAS239, a novel AfPIs targeting choline kinase alpha (ChoKα), and realized the goals of breast cancer imaging, antitumor therapy and monitoring choline metabolism in breast cancer. Osada et al. (65) took heat shock protein 90 (Hsp90) as the target and used the inhibitor SNX-5422 to connect the near-infrared dye with the PEG chain as the linker to image the target area of the subtype estrogen receptor-positive luminal invasive lobular carcinoma. Their study was representative of the use of imaging to detect a histological subtype that is difficult to diagnose early. This application reflects the advantages of inhibitor probe imaging at the molecular level, which can achieve subtype classification and higher sensitivity than traditional imaging examination (66). It is also possible to use heat shock protein inhibitors to target and inhibit the overexpression of heat shock proteins in tumor cells, thereby enhancing the efficiency of NIR photothermal therapy (67). In addition, there are applications such as surgery navigation and postoperative reconfirmation of the tumor area (57, 68). The design of these probes is based on an actual clinical problem rather than simple imaging and diagnosis of tumors, so they have a promising application prospect in the clinic.

This section mainly discusses the three key elements, inhibitors, dyes and linkers, and their novel applications in the design of NIR AfPIs, with MAO and PSMA inhibitors as examples. Each element may have a significant impact on the fundamental properties of the probe. When designing the structure, not only the properties of the three elements but also the interactions between them must be considered to improve the pharmacokinetics and avoid adverse effects such as decreased affinity caused by the increased steric hindrance.

## Radiolabeled AfPIs

According to the imaging principle, radiolabeled AfPIs can be classified into single-photon-emission computed tomography (SPECT) and positron emission tomography (PET) probes. Compared with SPECT, PET has a lower radiation dose and higher resolution and sensitivity, but the high costs limit its application in primary medical institutions (69). SPECT probes can provide longer image acquisition time due to a longer half-life (a few hours to a few days). Unlike PET, which emits two 511-eV photons, SPECT probes can emit photons with different energies, allowing multiple probes to be imaged simultaneously (70, 71). Our focus is on the imaging effect of radioactive probes based on inhibitors, and due to the differences in radionuclides, the design ideas of the probes will differ significantly. Radioactive elements commonly used in labeling inhibitors include nonmetallic **C**, **F**, **Br** and **I**, while metal elements include **Ga**, **Cu**, **Tc** and **Zr**. Depending on their isotopes, **Ga** and **I** can be used for PET or SPECT imaging.

### Nonmetallic radionuclide labels

Nonmetallic radionuclide labels can be introduced with a low influence on the affinity of inhibitors because nonradioactive carbon, nitrogen and oxygen atoms are inherently present in various biomolecules and compounds. As a result, compared with the nonlabeled inhibitors, only minimal changes occur in the chemical properties of the final probes. In the PET imaging [^11^C] NMS-E973 probe constructed by Vermeulen et al. (72), the carbon atoms on the methyl group of the Hsp90 inhibitor NMS-E973 (**19**) (73) were replaced with **^11^C** (**20**) to conduct *in vivo* melanoma imaging. The time of synthesis and purification should be limited to 2-3 half-lives to ensure the effectiveness of the radiolabeled APSMI (74), and the half-life of **^11^C** is short, which limits its clinical application. However, the introduction of **^11^C** generally does not change the pharmacological properties of the parent inhibitor, and it can be used to study the fate of the inhibitor *in vivo*. Brown et al. (75) used the **^11^C**-labeled focal adhesion kinase (FAK) inhibitor GSK2256098 to study the pharmacokinetics of parent inhibitor *in vivo* and compared the distribution of probes in normal brain and tumor tissues to study the impact of tumors on the blood-brain barrier. Yu et al. (76) labeled the transient receptor potential channel subfamily member 5 (TRPC5) inhibitor HC608 (**21**) to obtain **22** to study its metabolism *in vivo* and the effect of targeting TrpC5. Moreover, the half-lives of **^13^N** and **^15^O**, at 10 min and 2 min, respectively, are too short to be used for labeling inhibitors.

**Figure 6:**
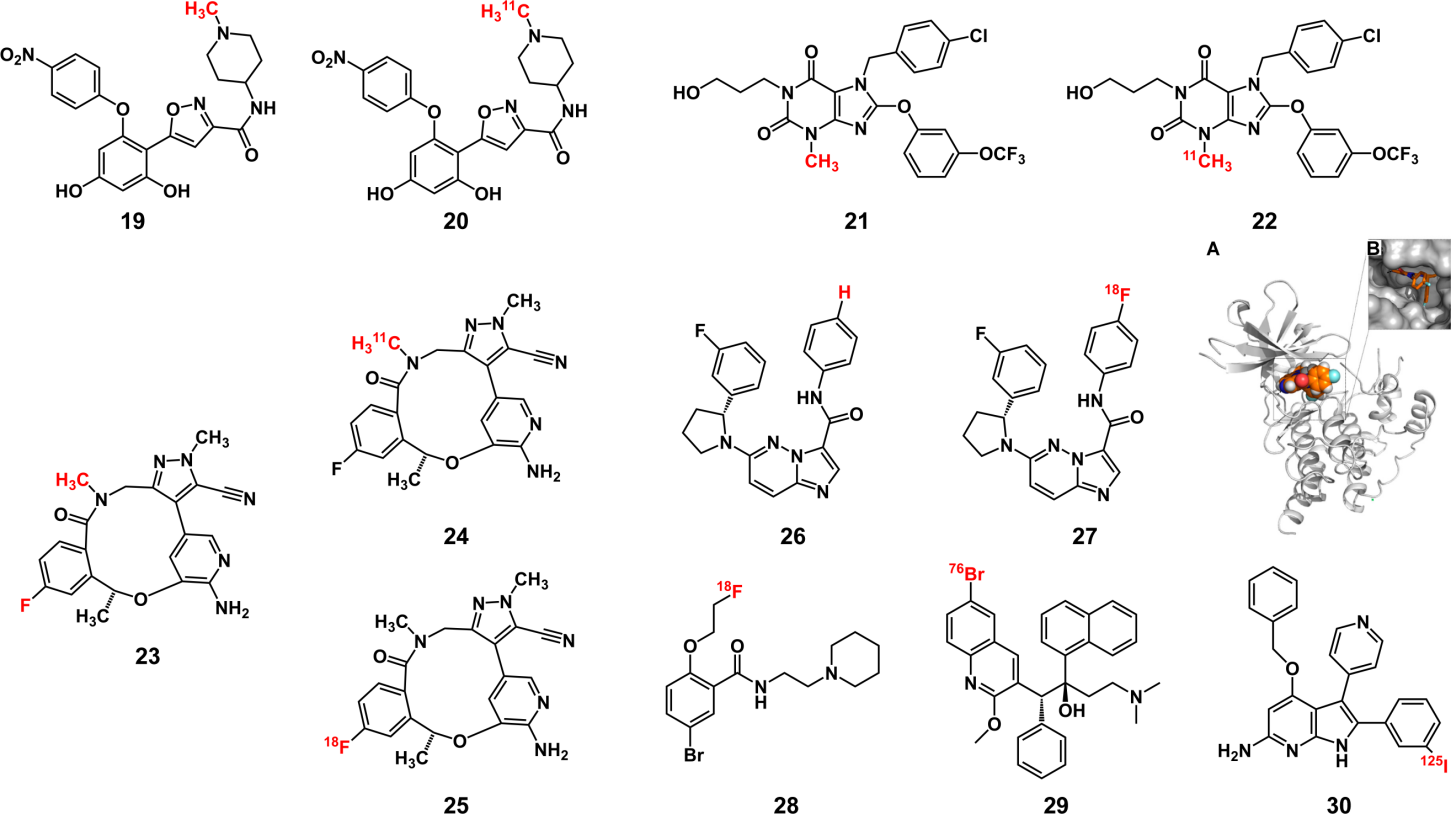
Some structures of radiolabeled AfPIs with nonmetallic radionuclide labels(Red: radionuclide labels). **(A, B)** The molecular docking result of [^18^F] labeled TrkA inhibitor with TrkA protein showed that the labeled inhibitor could bind with Trk. Reprinted with permission (84). Copyright 2018 American Chemical Society.

Probes labeled with halogen radionuclides have been widely used to diagnose tumors and metastases in the clinic. **^18^F**-labeled fludeoxyglucose (FDG) as a PET probe has been used particularly often (77), but due to the active glucose metabolism in the brain and inflammation, it still has limitations in tumor imaging (78–80). Such probes based on small-molecule inhibitors can reduce the false-positive rate because AfPIs can specifically bind to the target, and some of them have entered clinical trials (81). For inhibitors with fluorine in the structure, the loss of affinity caused by radiolabeling can be avoided, such as by replacing the fluorine atom (**24**) or carbon atom (**25**) on the benzene ring of the ROS1 inhibitor lorlatinib (**23**) (82). For inhibitors that do not contain fluorine atoms, **^18^F** can be substituted for a hydrogen atom or hydroxyl group through electrophilic or nucleophilic reactions, which will not cause significant steric hindrances because of their similar sizes. Additionally, the **C-F** bond formed is stronger than the **C-H** bond and thus is not easily destroyed in the body (83), which can decrease false positives in imaging. In the [**^18^F**] labeled tropomyosin receptor kinase (Trk) inhibitor (**27**) synthesized by Bernard-Gauthier et al. (84), the hydrogen atom on the benzene ring was replaced on the parent inhibitor (**26**), resulting in a loss of affinity. However, this loss is acceptable because it does not considerably affect the imaging effect of the probe (**Figures 6A, B**). Another method to add **F** to the noncritical area of the inhibitor, such as the PEG chain (**28**), which can also avoid the damage of steric hindrance to the affinity, can improve the metabolic kinetics of the probe and is conducive to the imaging effect (62, 85). However, it is necessary to verify the affinity of probes by molecular docking and affinity experiments.

Radiobromine and radioiodine are also commonly used labeling inhibitors. **^76^Br** (**29**) (86) and **^124^I** (87) are used for PET imaging and **^123^I/^125^I** (**30**) (88, 89) for SPECT Imaging. Although the steric hindrance of **I** and **Br** is greater than that of **F**, inhibitors can still be introduced through the abovementioned strategy, with a slight loss of affinity. In addition, these radionuclides exhibit a longer half-life than **^18^F**, facilitating the final synthesis of the AfPIs. When these halogen radionuclides are introduced, they may have greater affinity than the parent inhibitors (90), possibly ascribed to the electronegativity of the halogen radionuclides and the extra hydrogen bond formed between the radionuclides and the target receptor.

In general, introducing nonmetallic radionuclide labels to inhibitors to realize tumor imaging is relatively simple. Direct replacement of the original nonradioactive atoms or adding radionuclide with a linker, such as PEG, can avoid diminishing the affinity.

### Metallic radionuclide labels

Unlike nonmetallic radionuclide labels, metallic radionuclides cannot be directly introduced into the inhibitor, so the aid of a metal chelating agent is required. To allow the inhibitors to be labeled without considerably changing their physicochemical properties, bifunctional chelating agents are ideal candidates, which can conjugate with metal ions and inhibitors and can easily react with common functional groups (such as carboxyl, amino and alkyne/azide groups) on inhibitors to form stable covalent bonds (91).

Bifunctional chelating agents can be roughly classified into acyclic and macrocyclic, and the latter is more stable in complexation than the former (92). As a part of the linker in the probe, the chelating agent should be chosen after considering the following factors. The first requirement is that it does not affect the affinity of the original inhibitor and ensures that the ligand can bind to the target later. The design is the same as other AfPIs: The chelating agent cannot affect the critical binding group, and the change in steric hindrance needs to be considered. Second, the thermodynamic stability and kinetic inertness of the chelating agent should be ideal to avoid the release of metal ions to cause biological toxicity (93). During the synthetic process, the production of isomers should also be circumvented to avoid affecting the overall physicochemical properties of the probe. Based on the chelating agent, the metabolism of the probe can be improved by inserting hydrophobic/hydrophilic groups to achieve a careful balance, obtaining the optimal imaging effect. In addition, the insertion of PEG can improve water solubility and promote metabolism, which is governed by the same principle described above. The properties of metal radionuclides, such as size, shape and coordination number, also affect the choice of the chelating agent (94, 95). Therefore, when choosing a chelating agent, the nature of the metal radionuclides should be considered. Commonly used chelating agents for a given radionuclide are often not bad choices.

In summary, the design of radiolabeled AfPIs differs according to the kind of radionuclide. For nonmetal radionuclides, the atoms or groups in the parent inhibitor can be substituted directly, while for metal radionuclides, bifunctional chelating agents are warranted to reduce the loss of affinity. Regardless of the type, the main idea is to complete radionuclide labeling without lowering the affinity of the parent inhibitor while considering the metabolism and biological safety of the final product.

## Dual-modal AfPIs

The advantage of dual-modal probes is that they combine the two imaging technologies to take full advantage of each technique and offset their disadvantages, achieving the goals of high sensitivity and high resolution simultaneously. The most direct examples are PET/CT, SPECT/CT and PET/MRI, which use the anatomical information provided by CT or MRI technology to offset the insufficient spatial resolution of PET/SPECT, and these approaches have also been widely used in the clinic. PET/optical imaging (OI) or SPECT/OI can overcome not only the insufficient tissue penetration of fluorescent probes but also provide higher imaging resolution than PET and SPECT. Based on the connection of the PSMA inhibitor to the Cy3 fluorescent dye, Kommidi et al. (96) introduced **^18^F** through the click reaction at the distal end of the linker to achieve dual-modal imaging. PET imaging is helpful for preoperative planning, while fluorescence imaging can help surgery navigation for tumor resection and reconfirm the edge after surgery. Metal radionuclides can also be labeled on inhibitors with fluorescent dyes using bifunctional chelating agents. Baranski et al. (68) used Glu-urea-Lys-HBED-CC as the core structure to connect **^68^Ga** and various fluorescent dyes, and performed fluorescence-guided tumor resection in mice using a probe connected with IRDye 800CW. Near-infrared dyes provide a greater imaging depth for fluorescence imaging, making fluorescence-guided surgical resection possible, and deeper tumor tissues need to be positioned by PET before surgery. In addition to diagnosis and surgery navigation, PET/OI can be used to observe the administration and metabolism of the inhibitor by labeling the parent structure. Wang et al. (11) designed a PET/OI dual-modal dasatinib probe to compare the effect of convection-enhanced delivery on bypassing the blood-brain barrier and delivering it to glioma by intravenous administration. Fluorescence imaging overcomes the shortcoming that PET cannot monitor drug delivery at the cellular level. These applications are examples of solving clinical needs through the combination of radionuclide imaging and optical imaging. In addition, by adding functional groups, such as amino groups, to α-Fe_2_O_3_ nanoparticles, inhibitors and fluorescent dyes can be labeled to achieve MRI/OI dual-modal imaging (97).

As with single-modal AfPIs, the affinity, metabolism and tissue distribution should also be considered for dual-modal AfPIs. Conducting multiple labeling at the same time will inevitably cause more significant potential damage to affinity because it may alter more groups or cause greater steric hindrance, so additional dyes and radionuclide labeling should be as far as possible from the target area when the probe structure is being designed. Metabolism and tissue distribution need to be modified according to the *in vivo* performance of the core structure of probes. For example, Kimura et al. (98) used hydrophobic Cy5.5 dye to enhance tumor retention and reduce the impact of **^64^Cu** labeling on the imaging effect. Alternatively, increasing the number of sulfonate groups could improve the water solubility of the probe and switch the hepatobiliary to renal elimination, and a more concentrated signal at the tumor was obtained (99). Therefore, further structure modification can improve the metabolism and tissue distribution and eliminate the influence of multiple labels on the imaging effect of the probe.

Although much progress has been made in dual-modal imaging in recent years, the bimodal imaging probes including the dual-modal AfPIs still fall short of applicability in the clinic due to the limitations of the development of imaging instruments and software. However, these dual-modal or trimodal probes can provide more anatomical or functional information, and this considerable advantage is worthy of more research and development.

## Conclusion

The design and synthesis of AfPIs involve interdisciplinary research, and numerous issues need to be considered, including the affinity, distribution and pharmacokinetics *in vivo* of probes; the synthetic route; and translation to clinical applicability. It is necessary to perform molecular docking before designing probes to determine the effect of changes in steric hindrance and modification of moieties on their affinity. Moreover, the probe’s fate *in vivo* is crucial for imaging, and appropriate dyes and linkers can significantly improve the pharmacokinetic and imaging efficacy of the probe.

Over the last decade, there have been tremendous advances in the research of AfPIs. The AfPIs have been proved to have better specificity, smaller molecular weight, lower immunogenicity and faster targeting than protein and peptide probes. Although there are many reports on radiolabeled AfPIs, and some have entered into clinical trials, there is considerable room for improvement in NIR and dual-modal AfPIs, especially in the second NIR window. This challenge is related to the lack of suitable dyes and the greater difficulty of design and synthesis in the second NIR window probe. Therefore, problems such as developing new-generation inhibitors, NIR dyes and bifunctional chelators, improving quantum yield, and biological safety are still hindering the clinical application of AfPIs and are warranted to be solved. Finally, the probe is only a tool, and the ultimate objective is to solve medical needs. Hence, the final product should be convenient for clinical application in disease diagnosis or treatment.

In conclusion, it remains to be seen whether AfPIs can be applied in the clinic. However, with the development of more economical imaging instruments and new-generation inhibitors with fewer side effects and better selectivity, and the urgent need for more reliable detection methods and more efficient and safer treatment for cancer, AfPIs have broad prospects for cancer diagnosis and treatment monitoring.

## Author contributions

XY: initiating and writing the manuscript. ZW: initiating and writing the manuscript. XH: revising and editing the manuscript. AY: revising and editing the manuscript. All authors contributed to the article and approved the submitted version.

## Funding

This research is funded by the Open Competition Mechanism Science and Technology Project of Hubei Province (2022, NO.028).

## Conflict of interest

The authors declare that the research was conducted in the absence of any commercial or financial relationships that could be construed as a potential conflict of interest.

## Publisher’s note

All claims expressed in this article are solely those of the authors and do not necessarily represent those of their affiliated organizations, or those of the publisher, the editors and the reviewers. Any product that may be evaluated in this article, or claim that may be made by its manufacturer, is not guaranteed or endorsed by the publisher.
